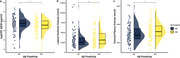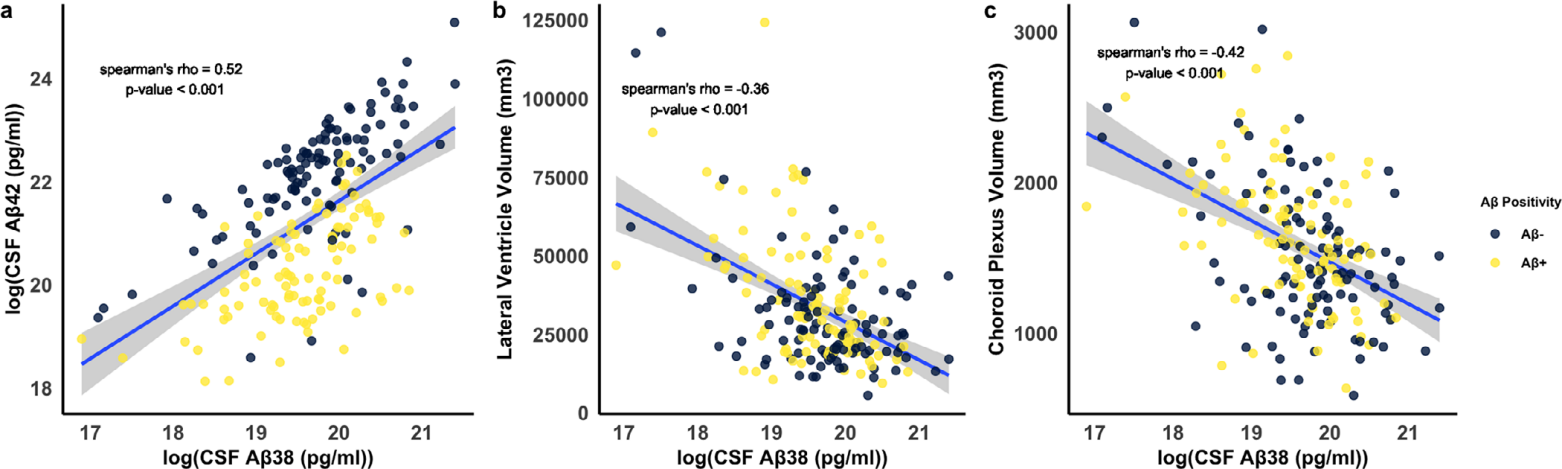# Ventricle Enlargement is Negatively Correlated with Aβ38 in human CSF

**DOI:** 10.1002/alz70856_107299

**Published:** 2026-01-08

**Authors:** Yansheng Zheng, Seyyed Ali Hosseini, Joseph Therriault, Brandon J Hall, Etienne Aumont, Arthur C. Macedo, Lydia Trudel, Yi‐Ting Wang, Nesrine Rahmouni, Stijn Servaes, Gleb Bezgin, Jaime Fernandez Arias, Tevy Chan, Jenna Stevenson, Kely Monica Quispialaya Socualaya, Wan Lu Jia, Thomas K Karikari, Tharick A Pascoal, Andrea L. Benedet, Nicholas Ashton, Kaj Blennow, Henrik Zetterberg, Paolo Vitali, Serge Gauthier, Gerhard Multhaup, Pedro Rosa‐Neto

**Affiliations:** ^1^ McGill University, Montreal, QC, Canada; ^2^ Translational Neuroimaging Laboratory, The McGill University Research Centre for Studies in Aging, Montréal, QC, Canada; ^3^ Montreal Neurological Institute, Montreal, QC, Canada; ^4^ Université du Québec à Montréal, Montréal, QC, Canada; ^5^ University of Pittsburgh, Pittsburgh, PA, USA; ^6^ Departments of Psychiatry and Neurology, University of Pittsburgh School of Medicine, Pittsburgh, PA, USA; ^7^ Department of Psychiatry and Neurochemistry, Institute of Neuroscience and Physiology, The Sahlgrenska Academy, University of Gothenburg, Mölndal, Sweden; ^8^ University of Gothenburg, Mölndal, Sweden; ^9^ The McGill University Research Centre for Studies in Aging, Montreal, QC, Canada; ^10^ Department of Neurology and Neurosurgery, and Department of Psychiatry, McGill Centre for Studies in Aging, McGill University, Montreal, QC, Canada; ^11^ Department of Pharmacology and Therapeutics, McGill University, Montreal, QC, Canada

## Abstract

**Background:**

Senile plaques in Alzheimer's disease (AD) primarily consist of Aβ42, while Aβ38 is a shorter isoform cleaved from Aβ42. Higher cerebrospinal fluid (CSF) Aβ38 levels have been linked to slower cognitive decline and a reduced risk of developing AD dementia. Ventricular enlargement, a CSF‐related abnormality in AD, is characterized by changes in lateral ventricle volume (LVV) and choroid plexus volume (CPV). This enlargement is associated with brain atrophy and cognitive decline, highlighting its relevance as a marker of disease progression.

**Method:**

A total of 204 individuals from the TRIAD cohort were analyzed including, 133 cognitively unimpaired (CU), 34 mild cognitive impaired (MCI) due to AD, and 37 AD dementia individuals. Aβ‐PET SUVR determined Aβ positivity. The levels of CSF Aβ38 and 42 were quantified by a novel nucleic acid linked immuno‐sandwich assay (NULISA). LVV and CPV in native space for each MRI were obtained using FreeSurfer (version 7.4.1). Spearman's correlation assessed the relationships between the levels of CSF Aβ38 and lateral ventricle volume, choroid plexus volume, and CSF Aβ42. Mann‐Whitney U test compared the levels of CSF Aβ38, LVV, and CPV across diagnostic group.

**Result:**

CSF Aβ38 levels were significantly lower in Aβ‐positive individuals compared to Aβ‐negative individuals (*p* < 0.05, Figure 1a). LVV and CPV were significantly increased in Aβ‐positive individuals (*p* < 0.05, Figure 1b and 1c). CSF Aβ38 levels were positively correlated with CSF Aβ42 levels (Spearman's rho = 0.52, *p* < 0.001; Figure 2a). CSF Aβ38 levels were negatively correlated with LVV (Spearman's rho = ‐0.36, *p* < 0.001; Figure 2b) and CPV (Spearman's rho = ‐0.42, *p* < 0.001; Figure 2c).

**Conclusion:**

The positive correlation between CSF Aβ38 and Aβ42 levels suggests a metabolic link at the molecular level, either through γ‐secretase cleavages or BACE1‐mediated degradation. The distinct pattern of Aβ38, LVV, and CPV in Aβ‐positive and Aβ‐negative groups suggests these biomarkers are linked with AD. This correlation indicates that the Aβ38/42 ratio could serve as a biomarker for enzymatic Aβ clearance. Finally, the negative correlation between CSF Aβ38 and LVV and CPV indicated that the macro‐CSF production is linked with Aβ clearance at micro level.